# Natural Course of Metabolically Healthy Overweight/Obese Subjects and the Impact of Weight Change

**DOI:** 10.3390/nu8070430

**Published:** 2016-07-15

**Authors:** Ruizhi Zheng, Chengguo Liu, Chunmei Wang, Biao Zhou, Yi Liu, Feixia Pan, Ronghua Zhang, Yimin Zhu

**Affiliations:** 1 Department of Epidemiology & Biostatistics, School of Public Health, Zhejiang University, Hangzhou 310058, Zhejiang, China; canprezrz@126.com (R.Z.); lyzju_2012@163.com (Y.L.); pfx19911119@163.com (F.P.); 2 Department of Endocrinology and Institute of Cardiovascular Diseases, Zhejiang Putuo Hospital, Zhoushan 316100, Zhejiang, China; Prylcg@mail.zsptt.zj.cn; 3 Tongxiang Center for Disease Control and Prevention, Tongxiang 314500, Zhejiang, China; chunmei328@sina.com; 4 Institute of Nutrition and Food Safety, Zhejiang Center for Disease Control and Prevention, Hangzhou 310000, Zhejiang, China; bzhou@cdc.zj.cn

**Keywords:** metabolically healthy, overweight and obese, weight change

## Abstract

Few studies have described the characteristics of metabolically healthy individuals with excess fat in the Chinese population. This study aimed to prospectively investigate the natural course of metabolically healthy overweight/obese (MH-OW/OB) adults, and to assess the impact of weight change on developing metabolic abnormalities. During 2009–2010, 525 subjects without any metabolic abnormalities or other obesity-related diseases were evaluated and reevaluated after 5 years. The subjects were categorized into two groups of overweight/obese and normal weight based on the criteria of BMI by 24.0 at baseline. At follow-up, the MH-OW/OB subjects had a significantly increased risk of developing metabolically abnormalities compared with metabolically healthy normal-weight (MH-NW) individuals (risk ratio: 1.35, 95% confidence interval: 1.17–1.49, *p* value < 0.001). In the groups of weight gain and weight maintenance, the MH-OW/OB subjects was associated with a larger increase in fasting glucose, triglycerides, systolic blood pressure, diastolic blood pressure and decrease in high-density lipoprotein cholesterol comparing with MH-NW subjects. In the weight loss group, no significant difference of changes of metabolic parameters was observed between MH-OW/OB and MH-NW adults. This study verifies that MH-OW/OB are different from MH-NW subjects. Weight management is needed for all individuals since weight change has a significant effect on metabolic health without considering the impact of weight change according to weight status.

## 1. Introduction

Obesity is a key risk factor for various diseases, including type 2 diabetes, cardiovascular disease (CVD), certain cancers, and musculoskeletal diseases [1]. However, it has been appreciated for many years that about 20% to 30% of obese adults exhibit fewer of these complications and do not meet all the criteria for metabolic syndrome [2]. Intense interest surrounds the “healthy” obese phenotype, which is defined as being obesity in the absence of metabolic syndrome (MetS), termed as metabolically healthy obesity (MHO) [3]. Despite the fact that there is no consensus on which criteria is preferable for defining MHO phenotype, longitudinal studies have suggested that this subtype of obesity are still at increased risk of diabetes and cardiovascular diseases [4,5,6,7]. Additionally, several studies have reported that MHO is not a permanent state and almost 30% to 50% of these individuals will convert to metabolically unhealthy status, with resultant increased cardiovascular risk [8,9]. Therefore, it has been speculated that the MHO subjects are not protected, but simply require additional time to develop adverse metabolic outcomes.

Weight change has a strong impact on metabolic status; however, several studies investigating the effects of weight loss by lifestyle intervention on metabolic status in the MHO subjects have yielded inconsistent results [10,11,12]. Most of these studies find that the MHO individuals obtain smaller improvements in MetS components from weight loss, compared with the metabolically abnormal obese (MAO) patients. Furthermore, it has been suggested that weight loss in postmenopausal MHO women may be unnecessary and paradoxically harmful given their favorable metabolic profiles [12]. Fabbrini et al. have evaluated the effect of a high-calorie diet on the metabolic status between the MHO and the MAO subjects [13]. The results demonstrate distinct differences in the response to weight gain. The MAO patients are predisposed to adverse metabolic effects of moderate weight gain, but not in the MHO subjects [13]. According to their conclusion, it can be speculated that the MHO subjects may maintain a healthy status in weight maintenance or even a little weight gain.

China has been experiencing an epidemic of obesity and metabolic disease in the last few decades; it has been estimated that almost 70% of Chinese adults are in the overweight and obesity category [14]. Furthermore, the Chinese population tend to accumulate fat intra-abdominally, and thus even lean subjects may have ectopic fat deposition and intra-abdominal obesity, accompanied with increasing cardiometabolic problems in overweight and obese subjects [15]. Our previous study found that 27.9% of obese participants are in metabolic health in the Chinese population [16]. However, little is known regarding the impact of weight changes on the metabolic profile for metabolically healthy overweight/obese subjects in Chinese population. Given this background, the aim of this study was to assess the metabolic response to 5-year weight change for metabolically healthy overweight/obese (MH-OW/OB) adults.

## 2. Materials and Methods

### 2.1. Subjects

During 2009–2010, the population aged over 30 and residing in the city of Tongxiang and Zhoushan in the Zhejiang province, China, were invited to participate in a health survey aimed at identifying risk factors for non-communicable diseases. A total of 3603 subjects participated in the prospective study, and data collected in 2009–2010 were used as the baseline for the present study. A clinical assessment was repeated 5 years later. This study was conducted according to the guidelines laid down in the Declaration of Helsinki and all procedures involving human subjects were approved by the Institutional Review Board of School of Medicine, Zhejiang University, Zhejiang, China (ethic approval code: ZGL201304-3). Written informed consents were obtained from all participants.

**Figure 1:**
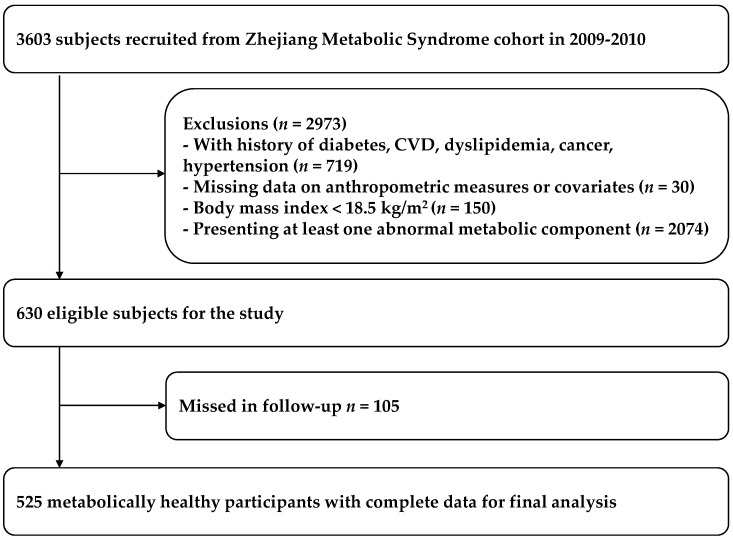
The flowchart of including subjects in the analysis.

According to the objective for the current study, those with a history of chronic diseases were excluded (e.g., diabetes, cardiovascular disease, dyslipidemia, cancer, hypertension) (*n* = 719). After exclusion of those with body mass index (BMI) < 18.5 kg/m^2^ (*n* = 150), and those who had missing anthropometric, metabolic detection data and questionnaire information (*n* = 30), 2704 participants were remained. Of these subjects, we selected those who had healthy metabolic status at baseline. Namely, all of the selected subjects had no metabolic abnormality of MetS definition of the International Diabetes Federation (IDF) criteria (waist circumference was not included in the definition of metabolic health because of collinearity with BMI), including elevated triglycerides (TG) (≥1.7 mmol/L), low high-density lipoprotein cholesterol (HDL-C) (men < 1.03 mmol/L, women < 1.29 mmol/L), elevated systolic blood pressure (SBP) (≥130 mm·Hg) or diastolic blood pressure (DBP) (≥85 mm·Hg), elevated fasting plasma glucose (FPG) (≥5.6 mmol/L) [17]. The selection yielded 630 subjects in metabolic health. After starting the study, 105 subjects (16.7%) were excluded for not attending a follow-up visit. Ultimately, 525 subjects were included in the analysis (Figure 1). General obesity was defined by BMI, which was recommended by the Working Group on Obesity in China (WGOC) [18]. There were 392 subjects in the normal weight category (BMI < 24.0 kg/m^2^), 98 in the overweight category (BMI 24 ~ 28 kg/m^2^) and 35 in the obesity category (BMI ≥ 28 kg/m^2^).

### 2.2. Clinical Measurements at Baseline and Follow-up

Follow-up measurements took place 5 years after baseline examination. Both at baseline and follow-up investigation, the same measurements were conducted. Nurses collected anthropometric data (weight, height) and blood pressure using standard protocols. Height and weight were measured on a scale, with the subjects wearing light clothing and without shoes. Waist circumference was measured twice after a normal expiration halfway between the lowest rib and the top of the pelvis. The mean of the two measurements was calculated. Blood pressure was measured in a sitting position with a mercury sphygmomanometer. SBP and DBP were reported as the average of three repeat measurements with 30-s intervals.

After a 12-h overnight fast, whole blood and serum samples were collected for each subject. All of the laboratory tests were conducted on fresh samples in an ISO-9002 quality-assured, core facility laboratory. Glucose was analyzed with a glucose oxidase method with the Beckman Glucose Analyzer (Beckman Instruments, Irvine, CA, USA). Biochemical variables including, TG, total cholesterol (TC), HDL-C and low density lipoprotein cholesterol (LDL-C) were determined using biochemical auto-analyzers (Hitachi 7060, Tokyo, Japan).

### 2.3. Covariates

At baseline, each participant had completed a self-reported questionnaire to determine ethnicity and lifestyle factors such as smoking behavior, alcohol intake, history of disease and medication use. Smoking and alcohol consumption status were regarded as positive when the participant was currently smoking and drinking; in the case of former or never, it was regarded as negative. International Physical Activity Questionnaire (IPAQ) (short vision) was used for the assessment of the average amount of time per week engaged in exercise activities [19]. The energy expended for each activity in metabolic equivalent (MET) minutes per week (MET-m/week) was calculated and summed.

### 2.4. Outcome Measurement

Follow-up measurements were conducted 5 years later. To determine the incident of metabolic abnormalities, the same threshold levels previously mentioned according to MetS definition of IDF criteria was used, including elevated TG, low HDL-C, elevated FPG and elevated SBP or DBP. Participants who used medication for either high blood pressure or high fasting glucose or a reduced HDL cholesterol or elevated triglycerides were defined as having the corresponding metabolic abnormalities. The occurrence of metabolically abnormal status was defined as presenting one or more metabolic abnormalities. Changes in these components were calculated as the values at follow-up minus the values at baseline. For each observation, weight change was calculated as the percent change between follow-up relative to the baseline ((weight at follow-up—weight at baseline)/weight at baseline × 100%). Weight change groups included weight loss, weight maintenance and weight gain, which were defined as percent change of <−3%, ≥−3% to ≤3% and >3%, respectively [20].

### 2.5. Statistical Analysis

We firstly classified the subjects into three groups by the BMI status at baseline (normal-weight, overweight and obesity). The software of Power Analysis and Sample Size (PASS) version 11 was used to calculate the statistical power for comparing the change of metabolic parameters (FPG, SBP, DBP, TG and HDL-C) among the groups of baseline BMI status. The results showed that statistical power ranged from 43% to 56%. Therefore, we combined the subjects in overweight and obesity. The subjects were classified as metabolically healthy normal weight (MH-NW) and metabolically healthy overweight/obese (MH-OW/OB). The statistical power increased to over 85%, which is enough to detect reasonable departures from the null hypothesis.

Continuous variables were checked for normality using the Kolmogorov-Smirnov test and the skewed variable (alanine aminotransferase) was log transformed. Categorical variables are presented as percentages. Chi-square tests were used for categorical variables. The comparisons of baseline clinical and anthropometry parameters between the groups were assessed using a 2-by-2 (body size-by-metabolic status at follow-up) ANCOVA, and Bonferroni correction was used to control for the inflation of type I error due to multiple comparisons.

Relationships between incident metabolic abnormalities were investigated with multivariable logistic regression analyses, and odds ratios (ORs) and 95% confidence intervals (95% CIs) were calculated with MH-NW subjects as the reference to assess the risk of developing metabolic abnormalities for MH-OW/OB. The OR and 95% CI were then used to compute risk ratio (RR) and 95% CI [21]. Models were adjusted for age, sex, smoking status, alcohol drinking status, physical activity, absolute weight change and the correspondingly metabolic parameters at baseline. Changes of metabolic parameters from baseline to follow-up were assessed by using mixed effects models with compound symmetry accounting for within-subjects correlation (multiple observation from a participant) with adjusting for the covariates previously mentioned.

We did not conduct sex-specific analyses because there were too few events in some subgroups to calculate stable risk estimates. A *p*-value < 0.05 was considered significant. All the statistical analysis was conducted using SAS software package (version 9.3; SAS institute, Cary, NC, USA).

## 3. Results

At baseline, the sample consisted of 630 individuals although loss to follow up resulted in a final analytic sample of 525 individuals. We checked for withdrawal bias by testing for differences in baseline variables between the participants who participated in follow-up measurements and those who did not participated in follow-up examinations. There were no differences in age, gender, metabolic parameters, BMI, smoking status, alcohol drinking status, and education level between the participated and non-participated individuals (Table S1).

At baseline, 392 participants were classified as MH-NW and 133 as MH-OW/OB. Baseline characteristics of the participants are presented in Table 1. The subjects in MH-OW/OB had larger values of baseline BMI and waist, and slightly higher DBP compared with MH-NW individuals.

After 5 years, the proportion of individuals in each weight change category was not significantly different between MH-NW and MH-OW/OB participants. The MH-NW subjects had greater increase in body weight, BMI and waist circumference than that of MH-OW/OB subjects. However, the MH-OW/OB subjects had more detrimental changes in all the metabolic parameters compared with the MH-NW counterparts (Table 1). Correspondingly, higher incidents of metabolic abnormalities were observed in the MH-OW/OB than MH-NW subjects. Compared with the MH-NW subjects, MH-OW/OB group was at a significantly elevated risk for developing pre-diabetes (RR = 2.01, 95% CI = 1.42–2.57, *p* < 0.001), high SBP (RR = 1.50, 95% CI = 1.11–1.91, *p* < 0.001), low HDL-C (RR = 1.69, 95% CI = 1.18–2.29, *p* < 0.001) and one or more metabolic abnormalities (RR = 1.35, 95% CI = 1.17–1.49, *p* < 0.001) with adjusting for age, gender, smoking status, alcohol drinking status, physical activity, weight change and correspondingly baseline metabolic parameters.

Both baseline BMI status and weight change contributed to the changes of metabolic parameters over time (both *p* < 0.01). No interaction of baseline BMI status by weight change category (*p* > 0.05) was noted for the changes of metabolic parameters. Table 2 presents the comparison of the paired-difference of the metabolic parameters between the MH-NW and MH-OW/OB subjects within each weight change category. In the weight loss group, there was no significant difference in the changes of these metabolic parameters between the MH-NW and MH-OW/OB adults. In the weight maintenance category, the MH-OW/OB subjects had significantly larger increase in TG (0.34 vs. 0.16 mmol/L, *p* = 0.040), SBP (12.4 vs. 7.4 mm·Hg, *p* = 0.020), DBP (5.6 vs. 1.9 mm·Hg, *p* = 0.001) and decrease in HDL-C (−0.21 vs. −0.08 mmol/L, *p* = 0.014) compared with MH-NW peers. In weight gain category, significantly more deleterious changes in FPG, SBP, DBP and HDL-C were observed in the MH-OW/OB subjects.

The MH-NW subjects with weight maintenance and weight gain had larger increase in SBP compared with the subjects in weight loss. Among the MH-OW/OB adults, weight maintainers had larger increase in DBP (5.6 vs. 1.1 mm·Hg, *p* = 0.003) and decrease in HDL-C (−0.21 vs. −0.11 mmol/L, *p* = 0.029) than that of the subjects in weight loss. Both the MH-NW and the MH-OW/OB subjects in the weight gain group lead to worse changes in metabolic parameters compared with the subjects in the weight loss group.

## 4. Discussion

This is, to the best of our knowledge, the first prospective study describing the characteristics of developing metabolic abnormalities in MH-OW/OB subjects in the Chinese population. In this prospective study, we found that the MH-OW/OB subjects had higher incidences of metabolic derangements compared with MH-NW counterparts as time passed. Our findings relating to stability in healthy individuals with excess fat over time were consistent with recently reported studies. Another prospective study in Spanish had shown that 30%–40% of healthy obese subjects had converted to the unhealthy status after 6 years of follow-up. Bobbioni et al. also examined the change of the metabolic status in the metabolically healthy overweight/obese individuals. At 3 years follow-up, the incidence of one or more cardiometabolic risk factors was 57.2% in the overweight/obese adults compared with 31.7% in the normal-weight subjects [22]. In the present study, the subjects in the overweight and obesity groups were positively correlated with higher incidences of metabolic abnormalities over time compared with the MH-NW subjects. This suggested that the MH-OW/OB individuals underwent more deterioration in metabolic change associated with excess fat. Our findings might explain the reason that the MH-OW/OB individuals had a higher risk for incident diabetes and cardiovascular events compared with that of MH-NW individuals [23]. Further longitudinal investigation of the sustainability and other predictors of the metabolic health subjects might better stratify the sub-type of obese individuals and provide potential intervention targets.

The beneficial effects of weight loss for the overweight and obese subjects have been well documented. However, intervention studies investigating the effect of weight loss on the metabolic status in MHO individuals had yielded contradictory results. Karelis et al. had carried out an intervention study in MHO subjects [12]. After 6-month energy-restricted diet, the MHO individuals exhibited significant reduction in body weight, accompanied with deterioration in insulin sensitivity [12]. Two similar studies observed no measureable effect of weight loss on inflammation levels in the MHO individuals [24,25]. Another two intervention studies also observed no significant improvements of the metabolic profiles in the MHO subjects [10,26]. However, a longer-term intensive lifestyle intervention including Mediterranean diet nutritional counselling and high-intensity interval training improved body composition and metabolic parameters in the MHO patients [27]. Similarly, three intervention studies found that energy-restricted diet and exercise intervention induced weight loss among the MHO subjects was associated with improvement in metabolic health status [28,29,30]. It was also suggested that the laparoscopic adjustable gastric banding was suitable for the morbidly obese individuals in metabolic health [31]. In the present study, under the natural conditions, the MH-NW and MH-OW/OB subjects in the weight loss group had similar changes in the metabolic parameters. They also presented better metabolic profiles than the subjects who gained weight.

Although weight maintenance literally implies no change in body weight, in free-living individuals, weight varies over time. The research by Forbes et al. showed that even with weight maintenance, adults lost about 1.5 kg of fat-free mass per decade [32]. In the present study, by using the definition of within ±3.0 percent change of baseline weight as weight maintenance, it showed that fasting plasma glucose, triglycerides, systolic blood pressure and diastolic blood pressure were significantly increased, while HDL-cholesterol decreased in weight maintainers. Naturally, long-term trends were superimposed upon the effects of aging in the longitudinal study. Truesdale et al. and Cui et al. compared the changes of the metabolic profiles between normal weight and obese subjects who maintained their weight based on the data from the Atherosclerosis Risk in Communities (ARIC) study [33,34]. However, they obtained opposite conclusions. The research of Truesdale et al. observed more favorable changes of metabolic parameters in the obese maintainers compared with the normal weight individuals, while Cui et al. reported reverse results. In the present study, the overweight and obese weight maintainers had more deleterious changes in the metabolic profiles than that of the normal weight maintainers. It suggested that the notion of metabolically healthy overweight/obese subjects should be used in caution, since weight maintenance and weight gain was associated with much more deleterious changes of metabolic conditions in MH-OW/OB adults compared with MH-NW.

Theoretically, MHO subjects were protected from the adverse metabolic effects of weight gain and increased adiposity, which had been proved by Fabbrini et al. [13]. They compared the metabolic response to a high-calorie diet intervention between the MHO and metabolically unhealthy obese subjects. Their results suggested that MHO phenotypes were protected against the adverse metabolic effects of weight gain by increased adipose tissue capacity for lipogenesis. However, plenty of studies have challenged the existence of a healthy obese phenotype by demonstrating that such subjects had higher risk of incident hypertension, type 2 diabetes, cardiovascular diseases than that of MH-NW individuals [24]. In line with these studies, our findings indicated that both the MH-NW and MH-OW/OB subjects had higher risk of advancing to metabolic abnormal status as a result of weight gain. A recent Mendelian randomization study concluded that increased adiposity had causal adverse effects on numerous risk markers for cardiovascular disease and type 2 diabetes in non-obese young adults [35]. Therefore, guidelines advising health care professionals to treat, monitor and prevent weight gain covered the population in all BMI would benefit from interventions on developing cardiometabolic risk factors.

Since the MHO phenotype was first described, many investigators have explored the characteristics that might distinguish these individuals from those with unfavorable metabolic status [36]. However, there was still lack of consensus on the definition of metabolic health. One study summarized that, up to now, there were at least 30 different definitions had been used to define a metabolically healthy phenotype in the literature [2]. Without specific and precise definition of metabolic health, we might not obtain an accurate risk estimate of metabolic diseases for MHO subjects. However, even if there would be a gold standard to accurately differentiate healthy and unhealthy subjects, it might still be invalid in the research of metabolic diseases. Since it has been generally accepted that overweight-to-moderately-obese individuals can be either metabolically healthy or unhealthy, it is not clear to what degree an individual could switch “categories” [37]. It might be efficient and convenient to operationally dichotomizing a continuous metabolic parameter above or below a certain threshold of interest so as to target persons who were at risk, but it would ignore the variability of metabolic status and other influencing factors during the follow up. Data from the present study showed that weight change had a strong impact on the developing cardiometabolic risk factors. Dozens of longitudinal studies had assessed the risk of developing type 2 diabetes, fatty liver and cardiovascular disease for MHO subjects [38,39]. However, few of these studies had taken account of the effects of weight change after stratifying by body size and metabolic status category [40].

There were several limitations to this study. The relatively small sample size made it impossible to stratify the subjects into three categories by BMI categories (normal weight, overweight and obese). Secondly, it has been demonstrated that inflammation was secondary to obesity in humans. However, we did not have the information of inflammation markers, such as C-reactive protein. The inclusion of inflammation as a criterion might modify the identification of metabolically healthy and abnormal individuals at baseline. We were aware that the results from this study could be varied if we used a different definition of weight change. Yet owning to the relatively small sample size in the present study, when using the cut-off of 5.0% for weight change classification, the sample size became much less concentrated in weight loss and weight gain categories, but the results were similar to our primary analyses.

## 5. Conclusions

MH-OW/OB is a relatively unstable condition and a considerable portion of these individuals will transition into unhealthy status at follow-up. Therefore, the potential benefits of differentiating the MHO and metabolically unhealthy obese phenotypes in clinical practice in the Chinese population appear limited. Our results suggest that weight gain and weight maintenance are strong indicators for advancing to metabolic abnormalities. With regards to BMI, there is plentiful evidence that, for the same category of BMI, Chinese subjects have higher percentage of total body fat and higher risk of cardiometabolic disease compared with white subjects. Therefore, even though obesity management consumes considerable labor and expense, weight management is needed for all individuals since weight change has a significant effect on metabolic health without considering the effect of weight change according to weight status.

## Figures and Tables

**Table 1:** Characteristics of the metabolically healthy normal-weight (MH-NW) and metabolically healthy overweight/obese (MH-OW/OB) subjects at baseline and the incidences of the metabolic abnormalities at follow-up.

Variables	MH-NW (*n* = 392)	MH-OW/OB (*n* = 133)	*p*-Value
Female (*n* (%))	203 (51.8)	87 (65.4)	0.006
Age (years)	55.0 ± 9.0	55.6 ± 7.2	0.513
BMI (kg/m^2^)	21.15 ± 1.77	25.95 ± 1.84	<0.001
Waist circumference (cm)	77.52 ± 5.60	87.58 ± 5.81	<0.001
SBP (mm·Hg)	115.0 ± 8.6	116.8 ± 8.1	0.066
DBP (mm·Hg)	73.5 ± 6.4	76.2 ± 6.3	<0.001
FPG (mmol/L)	4.85 ± 0.37	4.88 ± 0.38	0.555
TC (mmol/L)	4.95 ± 0.88	5.05 ± 0.91	0.121
TG (mmol/L)	0.93 ± 0.32	0.98 ± 0.34	0.059
HDL-C (mmol/L)	1.54 ± 0.33	1.50 ± 0.28	0.196
ALT (U/L) *	1.31 ± 0.17	1.33 ± 0.19	0.266
Current smoking (*n* (%))	135 (34.4)	37 (27.7)	0.121
Current drinking (*n* (%))	105 (26.8)	25 (18.8)	0.065
Percent in weight change (*n* (%))			
Weight loss	84 (21.4)	42 (31.6)	0.054
Weight maintenance	167 (42.6)	52 (39.1)	
Weight gain	141 (36.0)	39 (29.3)	
Changes in measurement			
Body weight (kg)	0.75 ± 3.93	−0.47 ± 6.07	0.007
BMI (kg/m^2^)	0.24 ± 1.53	−0.24 ± 2.26	0.005
Waist circumference (cm)	2.48 ± 6.15	0.49 ± 5.42	0.026
FPG (mmol/L)	0.42 ± 0.59	0.55 ± 0.49	0.018
TG (mmol/L)	0.18 ± 0.55	0.29 ± 0.57	0.037
SBP (mm·Hg)	7.7 ± 13.6	12.1 ± 13.2	0.004
DBP (mm·Hg)	2.4 ± 8.3	4.1 ± 8.4	0.042
HDL-C (mmol/L)	−0.11 ± 0.32	−0.20 ± 0.31	0.016
Developing metabolic abnormalities at follow-up (*n* (%))			
Pre-diabetes	82 (20.9)	52 (39.1)	<0.001
Hypertriglyceridemia	51 (13.0)	28 (21.1)	0.026
High SBP	115 (29.3)	57 (42.9)	0.004
High DBP	51 (13.0)	31 (23.3)	0.005
Low HDL-C	77 (19.6)	49 (36.8)	<0.001
Developing one or more	218 (55.6)	100 (75.2)	<0.001

Abbreviations: MH-NW, metabolically healthy normal-weight; MH-OW/OB, metabolically healthy overweight/obese. BMI, body mass index; SBP, systolic blood pressure; DBP, diastolic blood pressure; FPG, fasting plasma glucose; TC, total cholesterol; TG, triglycerides; HDL-C, high-density lipoprotein cholesterol; LDL-C, low-density lipoprotein cholesterol; ALT, alanine aminotransferase. * Data were log transformed. Data are expressed as means ± standard deviation and number (percentage).

**Table 2:** Comparison of 5-year change of the metabolic parameters between the subjects of MH-NW and MH-OW/OB and within weight change category.

Variables	Weight Loss (<−3%)		Weight Maintenance (≥−3% to ≤3%)		Weight Gain (>3%)	
	MH-NW (*n* = 84)	MH-OW/OB (*n* = 42)	MH-NW (*n* = 167)	MH-OW/OB (*n* = 52)	MH-NW (*n* = 141)	MH-OW/OB (*n* = 39)
ΔFPG (mmol/L)	0.40 ± 0.04	0.52 ± 0.06	0.39 ± 0.04	0.52 ± 0.08	0.52 ± 0.05 ^b^	0.77 ± 0.09 ^c,d^
ΔTG (mmol/L)	0.07 ± 0.05	0.17 ± 0.08	0.16 ± 0.05	0.34 ± 0.08 ^d^	0.27 ± 0.05 ^c^	0.35 ± 0.09
ΔSBP (mm·Hg)	4.7 ± 1.0	8.7 ± 1.9	7.4 ± 0.9 ^a^	12.4 ± 1.8 ^d^	10.0 ± 1.1 ^c^	15.7 ± 2.18 ^c,d^
ΔDBP (mm·Hg)	−0.1 ± 0.7	1.1 ± 1.0	1.9 ± 0.6	5.6 ± 1.1 ^a,d^	3.9 ± 0.7 ^c^	8.5 ± 1.4 ^c,d^
ΔHDL-C (mmol/L)	−0.07 ± 0.03	−0.11 ± 0.05	−0.08 ± 0.02	−0.21 ± 0.04 ^a,d^	−0.19 ± 0.02 ^c^	−0.27 ± 0.04 ^c,d^

Δ Absolute values between baseline and follow-up were expressed as mean ± standard error with adjustment for age, gender, smoking status, alcohol drinking status, and absolute weight change. Abbreviations: MH-NW, metabolically healthy normal-weight; MH-OW/OB, metabolically healthy overweight/obese; SBP, systolic blood pressure; DBP, diastolic blood pressure; FPG, fasting plasma glucose; TG, triglycerides; HDL-C, high-density lipoprotein cholesterol. ^a^ Significant difference between weight loss and weight maintenance within each baseline BMI category; ^b^ Significant difference between weight maintenance and weight gain within each baseline BMI category; ^c^ Significant difference between weight loss vs. weight gain within each baseline BMI category; ^d^ Significant difference between MH-NW and MH-OW/OB within each weight change category.

## Supplementary Materials

The following are available online at http://www.mdpi.com/2072-6643/8/7/430/s1, Table S1: The comparison of the baseline characteristics of the people participated in follow-up examinations and lost to follow up.
