# Supplementary material for: Natural Course of Metabolically Healthy Overweight/Obese Subjects and the Impact of Weight Change
Source: Nutrients. 2016 Jul 15;8(7):430. doi: 10.3390/nu8070430 (PMC4963906; doi:10.3390/nu8070430)
Supplement: Supplementary file 1 [file nutrients-08-00430-s001.docx]

**Supplementary Materials: Natural Course of Metabolically Healthy Overweight/Obese Subjects and the Impact of Weight Change**

Ruizhi Zheng, Chengguo Liu, Chunmei Wang, Biao Zhou, Yi Liu, Feixia Pan, Ronghua Zhang and Yimin Zhu

**Table S1.** The comparison of the baseline characteristics of the people participated in follow-up examinations and lost to follow up.

| **Characteristics** | **Participants *n* = 525** | **Nonparticipants *n* = 105** | ***p*-Value** |
| --- | --- | --- | --- |
| Age (years) | 55.1 ± 8.8 | 55.6 ± 7.2 | 0.835 |
| TC (mmol/L) | 4.83 ± 0.89 | 4.75 ± 0.72 | 0.091 |
| TG (mmol/L) | 0.95 ± 0.32 | 0.92 ± 0.27 | 0.083 |
| HDL-C (mmol/L) | 1.53 ± 0.32 | 1.48 ± 0.29 | 0.053 |
| LDL-C (mmol/L) | 2.70 ± 0.67 | 2.62 ± 0.56 | 0.208 |
| Body weight (kg) | 58.47 ± 8.67 | 59.12 ± 9.22 | 0.484 |
| BMI (kg/m^2^) | 22.36 ± 2.74 | 22.44 ± 2.85 | 0.790 |
| Waist (cm) | 80.37 ± 7.25 | 80.53 ± 6.65 | 0.540 |
| SBP (mmHg) | 115.5 ± 8.5 | 115.1 ± 9.2 | 0.580 |
| DBP (mmHg) | 74.1 ± 6.4 | 73.0 ± 6.5 | 0.802 |
| Gender (male) (*n* (%)) | 235 (44.8) | 52 (49.5) | 0.508 |
| Current smoking (*n* (%)) | 179 (31.4) | 36 (34.6) | 0.583 |
| Current drinking (*n* (%)) | 108 (20.6) | 17 (16.7) | 0.300 |
| High education level (*n* (%)) | 172 (32.8) | 39 (37.6) | 0.385 |

Abbreviations: TC, total cholesterol; TG, triglycerides; HDL-C, high-density lipoprotein cholesterol; LDL-C, low-density lipoprotein cholesterol; SBP, systolic blood pressure; DBP, diastolic blood pressure; BMI, body mass index. Data are expressed as means ± standard deviation and number (percentage).
